# The MIR-Domain of PbbHLH2 Is Involved in Regulation of the Anthocyanin Biosynthetic Pathway in ”Red Zaosu” (*Pyrus* *Bretschneideri* Rehd.) Pear Fruit

**DOI:** 10.3390/ijms22063026

**Published:** 2021-03-16

**Authors:** Xieyu Li, Fangxin Xiang, Wei Han, Bingqing Qie, Rui Zhai, Chengquan Yang, Zhigang Wang, Lingfei Xu

**Affiliations:** College of Horticulture, Northwest A&F University, Taicheng Road NO.3, Yangling 712100, Shanxi Province, China; junyouki@sina.com (X.L.); x_fangxin@foxmail.com (F.X.); 15664665857@163.com (W.H.); q1459151383@163.com (B.Q.); zhongdishaonian@sina.com (R.Z.); cqyang@nwsuaf.edu.cn (C.Y.); wzhg001@163.com (Z.W.)

**Keywords:** anthocyanin biosynthesis, MIR-domain, protein complexes, PbbHLH2, PbMYBs, pear fruit

## Abstract

The N-terminal of Myc-like basic helix-loop-helix transcription factors (bHLH TFs) contains an interaction domain, namely the MYB-interacting region (MIR), which interacts with the R2R3-MYB proteins to regulate genes involved in the anthocyanin biosynthetic pathway. However, the functions of MIR-domain bHLHs in this pathway are not fully understood. In this study, PbbHLH2 containing the MIR-domain was identified and its function investigated. The overexpression of *PbbHLH2* in ”Zaosu” pear peel increased the anthocyanin content and the expression levels of late biosynthetic genes. Bimolecular fluorescence complementation showed that PbbHLH2 interacted with R2R3-MYB TFs PbMYB9, 10, and 10b in onion epidermal cells and confirmed that MIR-domain plays important roles in the interaction between the MIR-domain bHLH and R2R3-MYB TFs. Moreover, PbbHLH2 bound and activated the dihydroflavonol reductase promoter in yeast one-hybrid (Y1H) and dual-luciferase assays. Taken together these results suggested that the MIR domain of PbbHLH2 regulated anthocyanin biosynthesis in pear fruit peel.

## 1. Introduction

Anthocyanins represent a major category of secondary metabolites found in many horticultural products [1,2,3]. Anthocyanin accumulation results in plant tissues presenting different colors [3]. In addition, anthocyanins play important roles in plant growth and development, as well as resistance to stresses, such as oxidation [4], light [4,5], and cold [6,7,8]. Anthocyanin is also involved in defending against pathogens [9] and attracting pollinators and seed dispersers [10]. They are also beneficial to human health [11,12,13]. Consuming anthocyanins may improve the body’s metabolism and energy balance, which aids in weight control, thereby reducing obesity risk [14]. Anthocyanins also have roles in fighting other diseases with their antibacterial [15] and antitumor [11,16] activities. Previous studies have indicated that anthocyanin could enhance eye and brain health and functions [11], effectively regulate blood pressure, blood lipids and blood glucose levels [17,18,19]. Additionally, anthocyanins play important roles in the prevention of cardiovascular and nervous system diseases [3,20].

Earlier studies have revealed that the anthocyanin biosynthetic pathway is composed by multiple enzymes classified as early biosynthetic genes and late biosynthetic genes (LBGs). The early biosynthetic genes include chalcone synthase and chalcone isomerase. The LBGs include dihydroflavonol reductase (*DFR*), anthocyanidin synthase (*ANS*)/leucoanthocyanidin dioxygenase, and UDP-glucoside: flavonoid glucosyltransferase (*UFGT*) [21,22,23,24].

Anthocyanins’ biosynthesis is regulated by transcription factors (TFs), such as MYBs and basic helix-loop-helixes (bHLHs). In *Arabidopsis thaliana*, *PAP1 (AtMYB75)*, *PAP2 (AtMYB90)*, *MYB113*, and *MYB114* are involved in anthocyanin accumulation [23,25,26]. In apple (*Malus* × *domestica*), *MYBA*, *MYB1*, and *MYB10* regulate the anthocyanin biosynthetic pathway [27,28]. In pear (*Pyrus bretschneideri* Rehd.), *MYB10* and *MYB10b* are involved in the anthocyanin biosynthetic pathway [29,30,31].

The bHLH proteins are a TF class in which each member contains a basic helix-loop-helix structural domain [32] that is important for the formation of the homodimers or heterodimers [33,34]. In plants, the more than 500 known bHLHs are divided into 26 subgroups [35]. Some bHLH proteins associated with the anthocyanin biosynthetic pathway have been identified in fruits, such as grapevine (*Vitis vinifera*), apple, strawberry (*Fragaria* × *vesca*), and pear [7,36,37,38,39]. The N-terminal interaction domain of IIIf bHLH TFs, also known as the MYB-interacting region (MIR) domain, was identified to interact with the R2R3-MYB domain proteins to regulate the anthocyanin biosynthetic pathway [40,41,42,43,44]. The bHLHs, as a subgroup of IIIf, plays important roles in regulating in anthocyanin biosynthesis in plants [21]. The IIIf bHLH TFs can interact with R2R3-MYB TFs and TTG1 (WD40) to form the MYB-bHLH-WD40 (MBW) ternary protein complex [45]. The MBW complex plays important roles in the regulation of LBGs in the anthocyanin biosynthetic pathway [21]. In the MBW complex, bHLH TFs determine the specificities of the recognized target gene promoter and the specificity of the activated transcriptional binding site [46]. Therefore, it is particularly important to explore the roles of bHLH TFs in plant anthocyanin biosynthetic pathways.

In this study, two bHLHs containing MIR-domain were identified as the positive regulators of anthocyanin biosynthesis in pear fruit. The functions of *PbbHLH2* in anthocyanin biosynthesis were investigated in pear fruit peel. The MIR-domain played essential roles in the interactions between PbbHLH2 and PbMYB9, 10, and 10b. The MIR-domain PbbHLHs interacted with PbMYBs to form complexes that accelerated anthocyanin biosynthesis by promoting the expression of *PbDFR*, *PbANS*, and *PbUFGT* in pear fruit. In addition, the MIR-domain PbbHLH2 independently induced anthocyanin accumulation and regulated anthocyanin biosynthetic genes expression. Thus, we found that a bHLH TF belonging to the IIIf subgroup, MIR-domain *PbbHLH2*, is involved in anthocyanin synthesis in pear fruit.

## 2. Results

### 2.1. Phylogenetic Analysis and Sequence Analysis of the Anthocyanin Related IIIf bHLH TFs in the Pear

**Figure 1:**
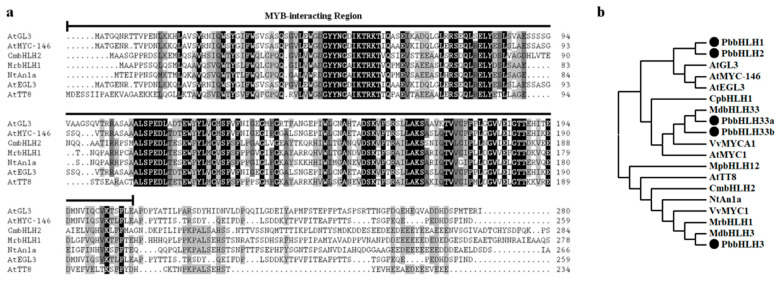
Analysis of IIIf bHLHs. (**a**) Multiple sequence alignment of the MYB-interacting region (MIR) domain of the IIIf bHLH transcription factors. Identical residues and conservative residues are marked in black and gray, respectively. The black line indicates the MIR. (**b**) Phylogenetic analysis of IIIf bHLHs from different species. The bHLH protein sequences of PbbHLHs were obtained from the NCBI. The gene accession numbers used are listed in Supplementary Table S1. At, *Arabidopsis thaliana*; Cm, *Chrysanthemum morifolium*; Cp, *Chimonanthus praecox*; Md, *Malus domestica*; Mp, *Marchantia polymorpha*; Mr, *Myrica rubra*; Nt, *Nicotiana tabacum;* Pb, *Pyrus bretschneideri*; Vv, *Vitis vinifera*.

Some bHLH proteins in the IIIf subgroup, such as *Arabidopsis thaliana* AtTT8, AtGL3, AtEGL3, AtMYC1, AtMYC-146, *Chrysanthemum morifolium* bHLH2, *Myrica rubra* bHLH1 and *Nicotiana tabacum* An1a, participate in the anthocyanin biosynthetic pathway [21,47]. By comparing the protein sequences of AtTT8, AtGL3, AtEGL3, AtMYC-146, CmbHLH2, MrbHLH1, NtAn1a and AtMYC1 of IIIf bHLHs, the MIR domain sequence was revealed (Figure 1a). The MIR-domain is essential for binding the R2R3-MYB to a transcription complexes [41,44]. The HMM model of the MIR-domain was constructed to screen the pear database (*Pyrus bretschneideri* Rehd.), and five bHLH proteins were identified and selected for further studies (Figure 1b). A phylogenetic tree containing the five candidate PbbHLH proteins and 14 IIIf subgroup bHLHs from different plants was constructed (Figure 1b). The multiple sequence alignment of IIIf proteins was presented in Supplementary Figure S1 (Supplementary Figure S1). We found that the PbbHLH1 and PbbHLH2 proteins, which are on a different branch from MdbHLH3 and MdbHLH33 proteins, regulated anthocyanin synthesis (Figure 1b). Therefore, we focused this study on the characterization of PbbHLH1 and PbbHLH2 function in anthocyanin biosynthesis in this study.

### 2.2. Expression Patterns of PbbHLH1 and PbbHLH2 Genes in Pear

**Figure 2:**
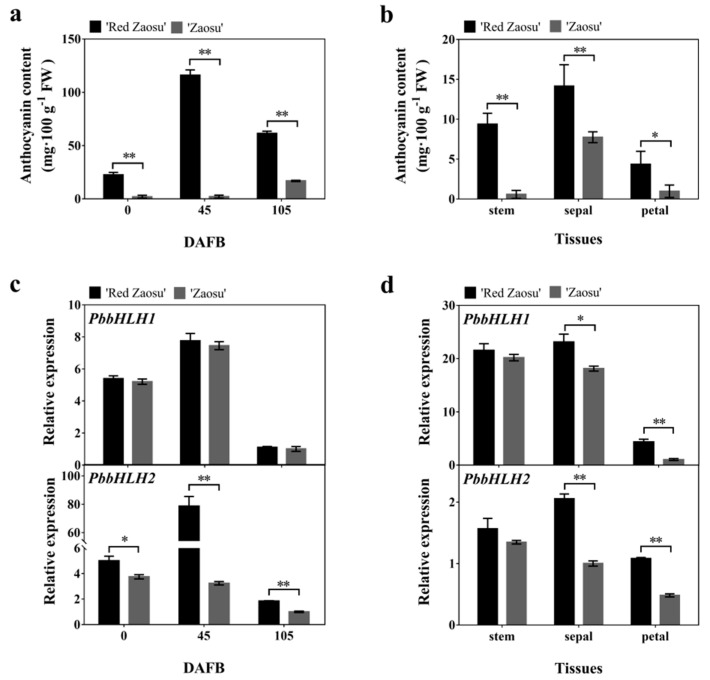
Expression patterns of bHLH transcription factors in different pear fruit tissues and at different developmental stages (**a**,**b**) The anthocyanin contents in pear fruit at different developmental stages (**a**) and in different pear fruit tissues (**b**); (**c**,**d**) *PbbHLH1* and *PbbHLH2* expression levels during different developmental stages (**c**) and in different tissues (**d**) of “Red Zaosu” and “Zaosu” pear. The significance levels of the differences were analyzed by t-test (* *p* < 0.05; ** *p* < 0.01). All data are from three biological replicates and are expressed as means ± SEs (*n* = 3). All tests were computed using SPSS (ver.20.0).

In order to explore the expression patterns of *PbbHLH1* and *PbbHLH2* genes in pear, the expression levels of *PbbHLH1* and *PbbHLH2* genes during three developmental stages of pear fruit and in three different tissues at 0 days after flower bloom (DAFB) were analyzed. The anthocyanin content in ”Red Zaosu” pear peel was higher than in “Zaosu” peel during each of the three stages analysed (Figure 2a), and in all the tested pear tissues at 0 days after flower bloom (Figure 2b). The expression level of *PbbHLH2* gene in “Red Zaosu” peel was significantly higher than in “Zaosu” peel in each of the three developmental stages (Figure 2c). Moreover, the expression levels of *PbbHLH1* and *PbbHLH2* genes in the sepals and petals of “Red Zaosu” were higher than in “Zaosu”. Additionally, the expression levels of *PbMYB9*, *10*, and *10b* genes in “Red Zaosu” peel were higher than in “Zaosu” peel (Supplementary Figure S2). Furthermore, *PbbHLH2* gene expression was positively correlated with the anthocyanin contents in different “Red Zaosu” pear fruit tissues and during different developmental periods (Supplementary Figure S3). Thus, *PbbHLH2* gene expression was correlated with anthocyanin accumulation in pear fruit. Therefore, we chose to further study *PbbHLH2* as an active regulator of anthocyanin biosynthesis.

### 2.3. PbbHLH2 up-Regulated Anthocyanin Accumulation in the Peel of Pear

**Figure 3:**
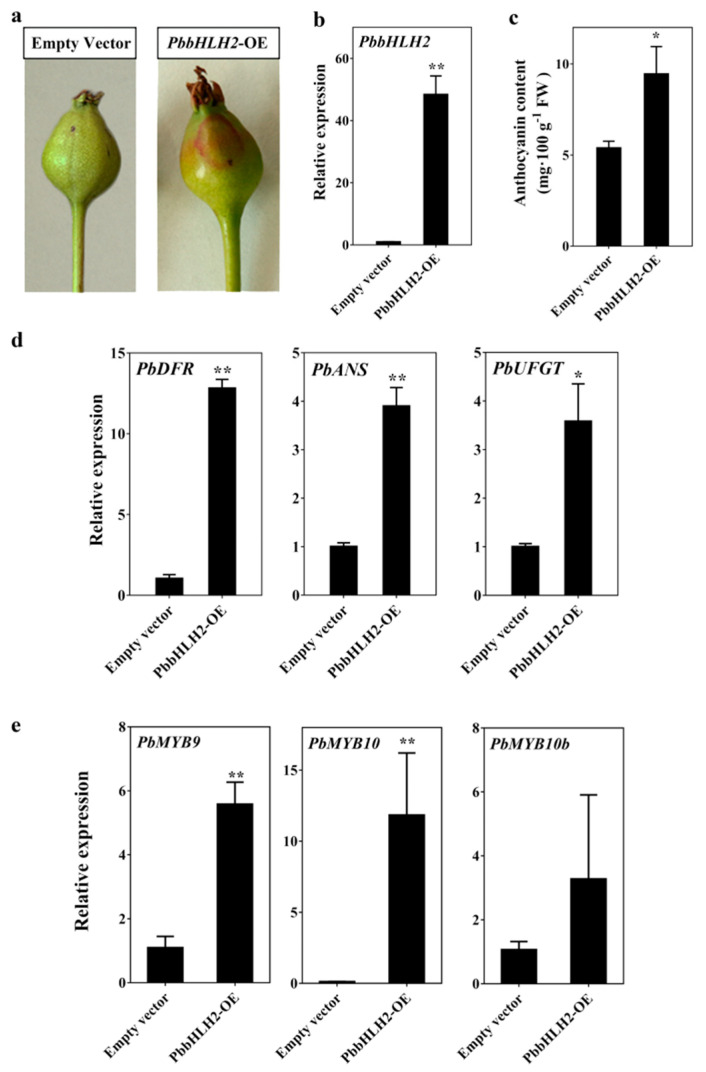
Anthocyanin patterns in pear fruitlets peel transiently overexpressing *PbbHLH2 (PbbHLH2*-OE). (**a**) Overexpression assay of *PbbHLH2* in “Zaosu” fruitlets peel; (**b**) The *PbbHLH2* gene expression levels in *PbbHLH2*-OE fruitlets peel; (**c**) The anthocyanin contents in *PbbHLH2*-OE fruitlets peel; (**d**,**e**) The expression levels of *PbDFR*, *PbANS* and *PbUFGT* (**d**) and of *PbMYB9*, *10*, and *10b* genes (**e**) in *PbbHLH2*-OE fruitlets peel. The significance levels of difference were determined by *t*-test (* = *p* < 0.05; ** = *p* < 0.01). All the data are from three biological replicates and are expressed as means ± SEs (*n* = 3). All the tests were computed using SPSS (ver.20.0).

In order to determine whether *PbbHLH2* gene is involved in anthocyanin biosynthesis, we overexpressed the *PbbHLH2* in “Zaosu” pear fruitlets peel. The effectiveness of the infection of the “Zaosu” fruitlets peel was verified by monitoring the GUS signal. The GUS reporter was used to monitor the gene expression patterns in the infected fruitlets peel (Supplementary Figure S4a). The peel of fruitlets overexpressing the *PbbHLH2* gene (*PbbHLH2*-OE) (Figure 3b) was redder than the one of fruitlets overexpressing the empty vector (Figure 3a). Moreover, the expression level in *PbbHLH2*-OE “Zaosu” pear fruitlets peel increased (Figure 3b), as did the anthocyanin content (Figure 3c). In addition, the transcript levels of *PbDFR*, *PbANS,* and *PbUFGT* genes were significantly increased in *PbbHLH2-OE* pear fruitlets peel (Figure 3d). The transcript levels of *PbMYB9*, *10*, and *10b* genes were increased in *PbbHLH2-OE* pear fruitlets peel (Figure 3e), as did the expression level of *PbGSTF12* gene (Supplementary Figure S4b). Thus, *PbbHLH2* gene promoted anthocyanin accumulation in pear fruitlets peel.

### 2.4. PbbHLH2 Gene was an Essential Part of the Anthocyanin Biosynthesis Pathway in the Pear Peel

**Figure 4:**
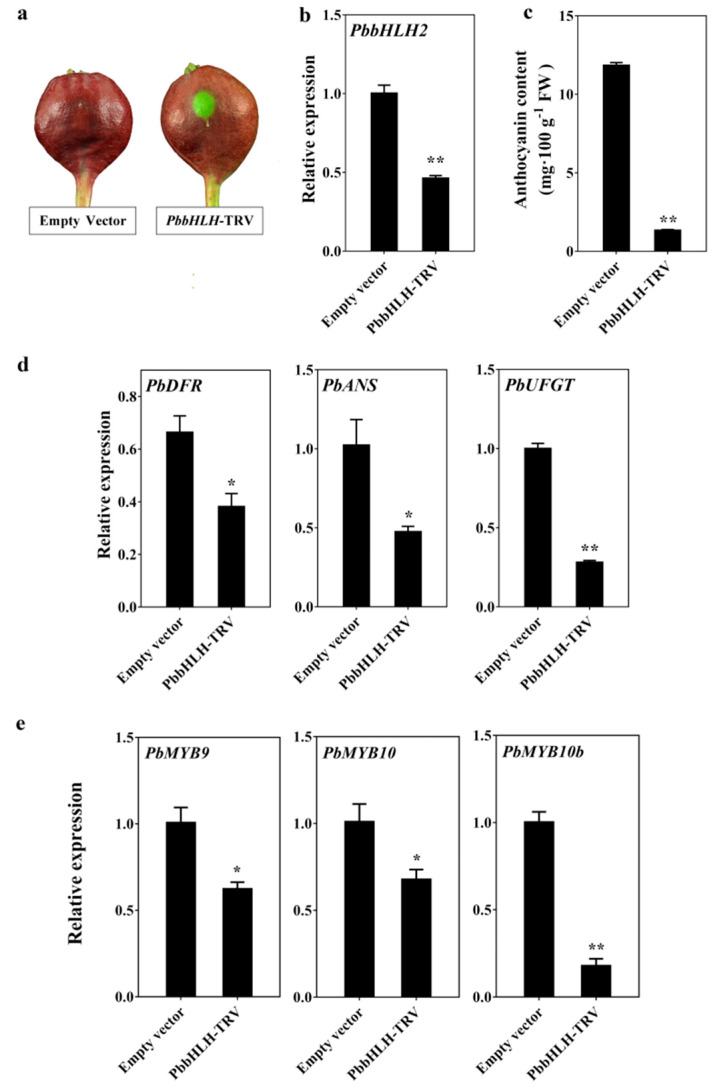
Anthocyanin patterns in transient *PbbHLH*-RNAi “Palacer” pear fruitlets peel. (**a**–e) VIGS assay (**a**); *PbbHLH2* expression levels (**b**); anthocyanin contents (**c**); *PbDFR*, *PbANS*, and *PbUFGT* expression levels (**d**); and *PbMYB9*, *10*, and *10b* genes expression levels (**e**) in transient *PbbHLH*-RNAi “Palacer” fruitlets peel. The significance levels of difference was analyzed by t-test (* = *p* < 0.05; ** = *p* < 0.01). All the data are from three biological replicates and are expressed as means ± SEs (*n* = 3). All the tests were computed using SPSS (ver.20.0).

To further verify the biological function of *PbbHLH2* gene in the anthocyanin biosynthetic pathway, the virus-induced gene silencing (VIGS) system was used to silence *PbbHLH2* gene in the peel of ”Palacer” pear fruitlets. The transient assay indicated that the *PbbHLH*-TRV fruitlets peel did not recover the red pigmentation around the injection holes (Figure 4a). The anthocyanin concentration in *PbbHLH*-TRV fruitlets peel significantly decreased (Figure 4c). In addition, the expression levels of *PbDFR*, *PbANS* and *PbUFGT* genes as well as those of *PbMYB9*, *10*, and *10b*, decreased compared with the empty vector (Figure 4d,e). Furthermore, the expression level of *PbGSTF12* in *PbbHLH*-TRV fruitlets peel decreased (Supplementary Figure S4c). Thus, *PbbHLH2* gene appears to play important roles in the anthocyanin biosynthetic pathway of pear fruit.

### 2.5. PbbHLH2 Interacts with PbMYB9, PbMYB10 and PbMYB10b via MIR-Domain

**Figure 5:**
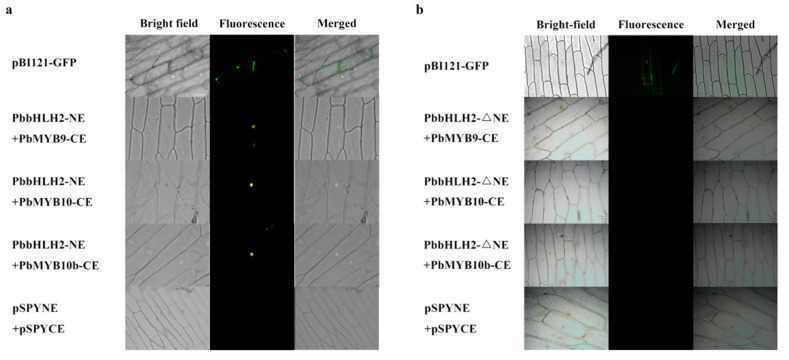
The interaction of PbbHLH2 and PbMYBs in onion epidermal cell. (**a**,**b**) The interaction of PbbHLH2 (**a**) and PbbHLH2-△NE, having a delete MIR-domain, (**b**) with PbMYB9, 10, and 10b in onion epidermal cells. The BiFC was observed using a fluorescence microscope (Axio Observer D1, Carl Zeiss Jena, Oberkochen, Germany).

To determine whether PbbHLH2 interacts with PbMYBs, a bimolecular fluorescence complementation (BiFC) analysis was performed using onion epidermal cells. PbbHLH2 protein interacted with PbMYB9, 10, and 10b proteins in the onion epidermal cell nucleus (Figure 5a). However, there was no fluorescence detected when PbbHLH2-△NE, having a delete MIR-domain, and PbMYBs were co-infiltrated in onion epidermal cells. These results indicated that the MIR-domain of PbbHLH2 was essential for interactions with PbMYB9, 10, and 10b (Figure 5b).

### 2.6. PbbHLH2 can Activate the Promoters of PbANS, PbDFR and PbUFGT in Pear Fruit Peel

**Figure 6:**
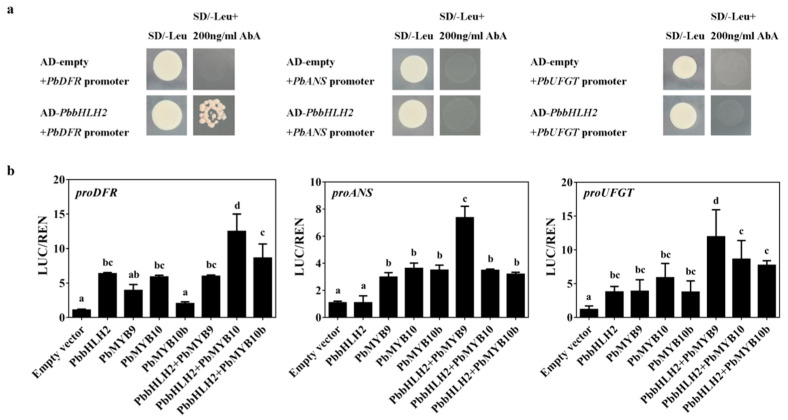
*PbbHLH2* interacted with *PbMYBs* to activate *PbDFR* and *PbUFGT*. (**a**) The interactions between the PbbHLH2 protein and the *PbDFR*, *PbANS*, and *PbUFGT* promoters as revealed by yeast one-hybrid assays. The yeast transformed with plasmid AbAi-promoters and plasmid AD-PbbHLH2 which grew on SD/-leu plate were used as positive controls; the yeast transformed with plasmid AbAi-promoters and empty plasmid AD which grew on SD/-leu + 200 ng/mL AbA plate were used as negative controls. (**b**) Transient dual-luciferase detections of *PbDFR*, *PbANS*, and *PbUFGT* promoters in *Nicotiana benthamiana* leaves. Different letters denote statistical significance (one-way ANOVA, *p* < 0.05). Values are means ± SDs, *n* = 3.

To investigate whether *PbbHLH2* gene binds the promoter regions of *PbANS*, *PbDFR,* and *PbUFGT* genes in pear, yeast one-hybrid assay (Y1H) was performed. The results showed that *PbbHLH2* directly bound the promoter of *PbDFR* gene (Figure 6a). When infiltrated into *Nicotiana benthamiana* leaves, *PbbHLH2* gene activated the *PbDFR* promoter but not the *PbANS* promoter (Figure 6b). When *PbbHLH2* was co-infiltrated with *PbMYB10*, the promoter of *PbDFR* was significantly activated (Figure 6b). When infiltrated with *PbMYB9* into *Nicotiana benthamiana* leaves, *PbbHLH2* gene can activate the *PbANS* and *PbUFGT* promoters (Figure 6b). Thus, *PbDFR*, *PbANS*, and *PbUFGT* were up-regulated when *PbbHLH2* was co-infiltrated with *PbMYBs*.

## 3. Discussion

Anthocyanins are important protective substances in plants that aid in resisting biotic and abiotic stresses [1,4,5,6,16]. The bHLH and MYB TFs play important roles in the anthocyanin biosynthetic pathway [48,49,50,51,52]. In *Arabidopsis thaliana*, AtGL3, AtEGL3, and AtTT8 are classified into the bHLH subgroup IIIf. Moreover, these three IIIf bHLH proteins contain a MIR-domain region and are involved in the anthocyanin biosynthetic pathway [53,54]. Until now, in bHLH TFs no definite MIR-domain had been identified as being involved in the anthocyanin biosynthetic pathway of pear fruit. In the present study, we identified two MIR-domain bHLH TFs, *PbbHLH1*, and *PbbHLH2*, in pear fruit. Both PbbHLH1 and PbbHLH2 are highly homologous with AtGL3/EGL3, which is known to regulate anthocyanin biosynthesis [53,55,56,57,58]. In some plants, such as tomato (*Solanum lycopersicum*) [55] and *Arabidopsis thaliana* [56,57,58], GL3 and EGL3 also play essential roles in anthocyanin biosynthesis. The expression patterns of genes may be used to infer their biological functions. Here, we detected high *PbbHLH2* gene expression levels in “Red Zaosu” pear fruit peel. This result was consistent with the anthocyanin contents of pear fruits (Figure 2). Therefore, we concluded that the biological function of *PbbHLH2* is related to the anthocyanin biosynthetic pathway. The overexpression *PbbHLH2* increased anthocyanin accumulations and anthocyanin structural gene expression levels in pear fruitlet peel (Figure 3). In agreement with this study, the transient overexpression of AtGL3 or AtMYC-146 can restore the production of anthocyanin production in a white-flowered *Matthiola incana* mutant [53]. Thus, *PbbHLH2* promoted anthocyanin biosynthesis in pear fruit peel.

Both *PbMYB9* and *PbMYB10b* are involved in the anthocyanin biosynthetic pathway [31]. In *Arabidopsis thaliana*, the MYB TFs *PAP1*, *PAP2*, *AtMYB113,* and *AtMYB114* act as positive regulators of anthocyanin accumulation [23,25,26]. Previous studies have shown that the MdMYB10 is autoregulated in red apple [59,60]. MdbHLH3 may not regulate the activation of MdMYB10 promoter [60], but MdbHLH3 may interact with MYB9, MYB10 and MYB110 to activate the MYB10 promoter in apple [59,60,61]. Therefore, the expression of MYBs is not completely consistent with the expression of bHLHs in pear fruit (Supplementary Figure S2). In *Arabidopsis thaliana*, the anthocyanin biosynthesis is regulated by the MBW protein complex through the transcriptional regulation of structural genes [25,62,63,64]. In our study, the expression levels of *PbMYB9*, *10*, and *10b* gene were affected by *PbbHLH2* gene in transient assays in pear fruit (Figure 3 and Figure 4). This suggests that *PbbHLH2* may form a dimeric structure with MYB. In our study, the MIR-domain was identified in the N-terminal of PbbHLH2 protein (Figure 1). We showed that the MIR-domain of PbbHLH2 protein interacts with PbMYB9, 10, and 10b, which are activators in the anthocyanin biosynthetic pathway. In addition, PbMYB9, 10, and 10b cannot interact with the PbbHLH2 when it lacks the MIR-domain. This indicates that the MIR-domain is essential for the interactions between bHLH2 and MYBs. Our study is in substantial agreement with the previous reports [44,45].

The earlier studies indicated that the MIR domains of IIIf bHLH members are indispensable for the interactions with R2R3-MYB TFs [43,44,65]. Both the bHLH domain and ACT-like domains form specific dimers that regulate the flavonoid biosynthetic genes [66,67]. The WD40/AD is an interaction site for WD40 and/or the RNA polymerase II through the acidic domains in bHLH proteins [68]. And MYB factors are involved in the anthocyanin biosynthetic pathways of some plants [49,50,62,69]. According to this study, the MIR domains in bHLHs interacted with MYBs to form dimers and affected LBGs expression levels in pear. Taken together, our results showed that the PbbHLH2 interacts with PbMYB9, 10, and 10b through the MIR-domain to form a transcription complex in pear fruit peel. Moreover, our study indicated that the MIR-domain is essential for the formation of bHLH-MYB protein complexes.

Previous studies have shown that the LBGs in anthocyanin biosynthesis are regulated by an MBW complex that consists of an R2R3-MYB, a subgroup IIIf bHLH TF and a WD40 repeat protein. For example, in *Arabidopsis*, R2R3-MYB TFs (PAP1, PAP2, MYB113, or MYB114), bHLH TFs (TT8, GL3 or EGL3) and the WD40 protein TTG1 can form the MBW transcriptional activator complex needed to regulate anthocyanin biosynthesis [25]. In *Paeonia suffruticosa*, PsbHLH1 could increase the transcription expression levels of *PsDFR* and *PsANS* by directly binding their promoters [70]. In the present study, we found that the PbbHLH2 directly bound to the promoter of *PbDFR* and induced the gene’s transcriptional activation (Figure 6).

In cornflower (*Centaurea cyanus*), CcbHLH1 interacts with CcMYB6–1 to form a complex protein that up-regulates the expression of *CcF3H* and *CcDFR* in the anthocyanin biosynthetic pathway [48]. In strawberry fruit, the expression of *FvDFR* is activated by the formation of heterodimers between FvHY5 and FvbHLH9 [71]. Our results were consistent with these previous studies. When the *PbbHLH2* was co-infiltrated with *PbMYB10*, *PbDFR* was activated in *Nicotiana benthamiana* leaves. Although the *PbbHLH2* did not bind to the promoter of *PbANS*, it induced the activation of *PbANS* promoter when co-infiltrated with *PbMYB9* into *Nicotiana benthamiana* leaves (Figure 6). Both the *PbMYB9* and *PbMYB10*, but not *PbMYB10b,* bind the *PbUFGT* promoter region [31]. Here, we found that the co-expression of *PbbHLH2* and *PbMYB9* induced *PbUFGT* expression. Therefore, *PbbHLH2* overexpression increased *PbANS* and *PbUFGT* expression levels in “Zaosu” pear fruit. Taken together, our results suggested that PbbHLH2 forms a bHLH-MYB protein complex through the MIR-domain and plays important roles in the anthocyanin biosynthetic pathway of pear fruit.

## 4. Materials and Methods

### 4.1. Plant Treatment and Growth Conditions

The fruit of “Zaosu” (*Pyrus bretschneideri* Rehd.), “Red Zaosu” (*Pyrus bretschneideri* Rehd.) and “Palacer” (*Pyrus communis* L.) from a commercial orchard in Mei County, Baoji, China, were selected as experimental materials in 2017. The “Red Zaosu” pear (*P. bretschneideri* Rehd.) is a bud sport of “Zaosu” pear. The regulatory mechanism of anthocyanin biosynthesis in “Red Zaosu” and “Zaosu” has been studied [31]. The “Palacer” had been used to transient assays in anthocyanin study [31]. The fruit of ”Palacer” was selected about 40 days after flower blossom (DAFB) and bagged for 30 days until the red pigment completely faded. The fruit of “Zaosu” and “Red Zaosu” were harvested at 0, 45, and 105 DAFB, respectively. The stem, sepal, and petal of “Zaosu” and “Red Zaosu” were harvested at 0 DAFB. The tissues of harvested fruit were frozen in liquid nitrogen and stored at −80 °C for the subsequent measurements of anthocyanin concentrations and RNA extraction.

*Nicotiana tabacum* plants were grown in a growth room with a photoperiod of 16/8 h (light/dark) at 22 °C. The transformation was performed with *Agrobacterium tumefaciens* strain EHA105 (Tolo Biotech., Shanghai, China) after the plant had at least six leaves.

### 4.2. Isolation of bHLH Genes and Their Phylogenetic Analysis

The sequences of bHLH proteins were isolated from the pear database [29]; (https://www.peargenome.njau.edu.cn/, accessed on 13 October 2017). The phylogenetic analysis was performed using the Neighbor-Joining method with a JTT model and a bootstrap test using the MEGA 7.0 program [72]. The GenBank accessions of the functionally labelled *bHLH* genes were listed in Supplementary Table S1. The complete coding DNA sequences (CDS) of candidate bHLH TFs and MYBs were cloned from “Red Zaosu” genomic DNA using PrimeSTAR Max Premix (TaKaRa, R045A, Dalian, China) with gene-specific primers (Supplementary Table S2).

### 4.3. RNA Isolation and Expression Analysis Using qRT-PCR

The total RNA was extracted from pear peel using the RNAprep Pure Plant Kit (Tiangen, DP441, Beijing, China). The RNA concentration and quality were detected by UV spectrophotometry and by running a 0.8% agar gel, respectively. In total, 1 μg of total RNA was reverse transcribed to cDNA using the PrimeScript RT reagent kit with gDNA Eraser (TaKaRa, Dalian, China). The primers used for qRT-PCR were designed with Oligo7 software [73] and synthesized by AuGCT Biotechnology Synthesis Lab (Beijing, China). The primers for actin, anthocyanin biosynthetic genes and candidate *bHLHs* and *MYB*s are described in Supplementary Table S2.

### 4.4. Transient Assays in Pear Fruit

The complete CDS of *PbbHLH2* was cloned into the multiple cloning sites (MCS) (BamHI-HindIII) of pGreenII 62-SK vector (*PbbHLH2-OE*, Supplementary Figure S5a) [1]. The *Agrobacterium tumefaciens* strain EHA105 containing *PbbHLH2*-*OE* was grown in Luria–Bertani solid medium (Oxiod, 81 Wyman Street, Waltham, MA, USA) at 28 °C. After 2 days of culture, the *Agrobacterium* was collected and re-suspended in infiltration buffer (10 mM MgCl_2_, 10 mM MES, pH 5.6, 200 mM acetosyringone), and shaken for 3–4 h at room temperature. The OD600 of *Agrobacterium* was adjusted to 0.8 with infiltration buffer and then injected into pear fruitlets. The fruit was harvested 3 days after injection.

The 400–600 bp fragments of *bHLH2* were inserted into the MCS of the pTRV2 VIGS vector (Supplementary Figure S5a). The constructed plasmid was transformed into *Agrobacterium* strain EHA105. The protocols of *Agrobacterium* culture and the injection of pear fruit were the same as above.

### 4.5. Anthocyanin Content Measurements

The content of total anthocyanin in red skin pear fruitlets was measured by pH differential method [74]. In this experiment, we used a previously reported method with slight modifications [74]. The 0.2 g sample was frozen and powdered in liquid nitrogen, and then 1.5 mL of 1% HCL-methanol extract was added. PVP K30 (Sigma, St. Louis, MO, USA) was added to the sample during grinding to prevent browning. After centrifugation at 4 °C and 12,000 rpm for 5 min, the supernatant was transferred separately to two clear tubes for dilution. One was diluted with 0.025 M potassium chloride buffer (pH 1.0), and the other with 0.4 M sodium acetate buffer (pH 4.5). These solutions were placed in the dark at room temperature before the absorbance values were measured synchronously at 520 nm and 700 nm using the Microporous plate spectrophotometer (Multiskan GO; Thermo Scientific, Waltham, MA, USA).

### 4.6. Dual-Luciferase Assay

The promoters of *PbANS*, *PbDFR*, and *PbUFGT* were amplified using PrimeSTAR Max Premix (TaKaRa, R045A) from “Red Zaosu” genomic DNA and gene-specific primers (Supplementary Table S2). These promoters were cloned into the HindIII and BamHI sites within the dual-LUC plasmid pGreenII 0800-LUC (Supplementary Figure S5b) [75]. The full-length CDS sequences of *PbbHLH2* and *PbMYBs* were cloned into the MCS (BamHI-HindIII) of the pGreenII 62-SK binary vector [75].

Each of these recombinant plasmids and the pSoup helper plasmid [75] were transferred individually into the *Agrobacterium* strain EHA105. EHA105 containing *PbbHLH2*-SK or/and *PbMYBs*-SK were separately mixed with *PbDFR* promoter-LUC, *PbANS* promoter-LUC or *PbUFGT* promoter-LUC at 1:1 ratio before infiltration into 4-week-old *N. benthamiana* leaves. The ratio of firefly luciferase to Renilla luciferase enzyme activities was analyzed using a Dual-Luciferase Reporter Assay System (Promega, Madison, WI, USA) with a Full Wavelength Multifunctional Enzyme Labelling Instrument (Infinite M200pro, TECAN, Männedorf, Switzerland). Proteins were extracted using 1 × PBS. Three independent experiments were carried out with at least four biological replicates per experiment.

### 4.7. Bimolecular Fluorescence Complementation (BiFC)

The CDS of candidate *bHLH*s were cloned into pSPYNE (named *PbbHLH2-YNE*), and the CDS of *PbMYBs* were fused into pSPYCE (named *PbMYB9-YCE*, *PbMYB10-YCE*, and *PbMYB10b-YCE*). The CDS of *PbbHLH2* without MIR-domain was cloned into pSPYNE (named *PbbHLH2-ΔNE*) (Supplementary Figure S5c). Then, the constructed plasmids were transformed into *Agrobacterium* strains (EHA105). BiFC assays were performed by the co-transfection of *Agrobacterium* harboring components of *PbbHLH2-YNE* and *PbMYBs-YCE* into onion epidermal cells [76]. The *Agrobacterium* contained P19 helper plasmid was mixing with the *PbbHLH2-YNE* and *PbMYBs-YCE* before infiltration. The pBI121-GFP plasmid was used as a positive control in this experiment. The onion epidermal tissues were cultured on Murashige & Skoog solid plates at 22 °C in darkness. The fluorescence of BiFC was collected using a fluorescence microscope (Axio Observer D1, Carl Zeiss Jena, Oberkochen, Germany).

### 4.8. Yeast One-Hybrid (Y1H) Assay

The Y1H screening was performed in terms of the Matchmaker Gold Yeast One-Hybrid System Kit (Clontech, Mountain View, CA, USA), as recommended by the manufacturer. The assay used the yeast strain Y1HGold, which is unable to grow in the selective synthetic dextrose medium (SD) absence of uracil. The pAbAi-baits were constructed by inserting the 800 bp fragments of the structural genes’ promoters into the pAbAi vector (Supplementary Figure S5b). The pAbAi-baits were linearized and transformed into Y1HGold cells. Meanwhile, the complete CDS of the *PbbHLH2* was cloned into pGADT7 vector to give the AD-prey vectors and then transformed into Y1HGold cells. After 3–4 days, these yeast strains were tested on a selective plate medium.

## 5. Conclusions

**Figure 7:**
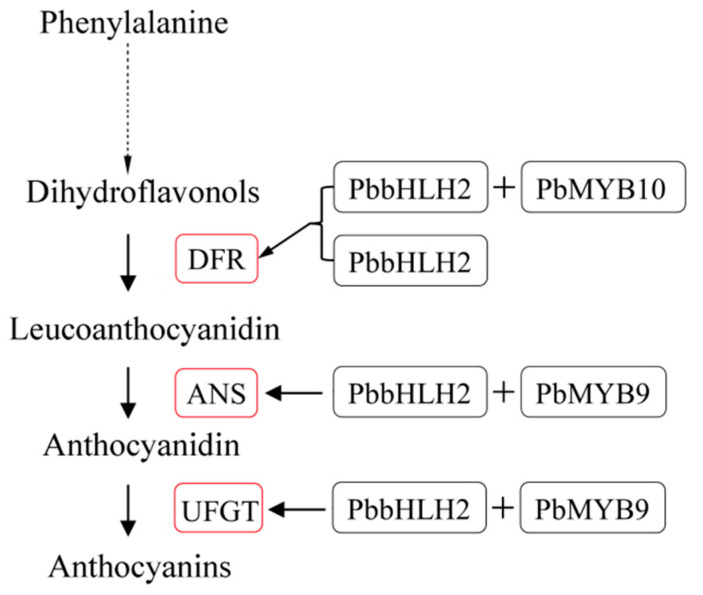
Proposed model for PbbHLH2 physical interaction, with or without PbMYB9 and PbMYB10, in the regulation of the anthocyanin biosynthetic pathway of pear. PbbHLH2 with or without PbMYB10 binds the promoter of *PbDFR* and up-regulates the gene’s expression. PbbHLH2 interacts with PbMYB9 to bind the *PbANS* and *PbUFGT* promoters and activate the genes’ expression. The red boxes indicate up-regulation.

On the basis of our results, a working model describing the function of the MIR-domain *PbbHLH2* in the anthocyanin biosynthetic pathway was proposed (Figure 7). The model was established using the known anthocyanin pathway with slight modifications [77,78,79]. The *PbbHLH2* gene independently regulates the *PbDFR* expression to participate in the anthocyanin biosynthetic pathway of pear fruit. Moreover, its encoded protein also forms complexes with PbMYB9 or 10. The protein complexes are involved in the anthocyanin biosynthetic pathway through the transcriptional regulation of *PbDFR*, *PbANS*, and *PbUFGT* in pear fruit.

## Data Availability

The data presented in this study are available in the article or supplementary material.

## Supplementary Materials

The following are available online at https://www.mdpi.com/1422-0067/22/6/3026/s1.

## Abbreviations

ANS	Anthocyanidin synthase
bHLH	Basic helix-loop-helix
BiFC	Bimolecular fluorescence complementation
CDS	Coding DNA sequence
DAFB	Days after flower bloom
DFR	Dihydroflavonol 4-reductase
HAS	Hours after sunrise of day 1
LBGs	Late biosynthetic genes
MBW	MYB-bHLH-WD40 ternary protein complex
MCS	Multiple cloning sites
MIR	MYB-interacting region
OE	Overexpression
RVE	REVEILLE
SD	Selective synthetic dextrose medium
TFs	Transcription factors
UFGT	UDP-glucoside: flavonoid glucosyltransferase
VIGS	Virus-induced gene silencing
Y1H	Yeast one-hybrid assay
