# Supplementary material for: The MIR-Domain of PbbHLH2 Is Involved in Regulation of the Anthocyanin Biosynthetic Pathway in ”Red Zaosu” (Pyrus Bretschneideri Rehd.) Pear Fruit
Source: Int J Mol Sci. 2021 Mar 16;22(6):3026. doi: 10.3390/ijms22063026 (PMC8002321; doi:10.3390/ijms22063026)
Supplement: Supplementary file 1 [file ijms-22-03026-s001.zip › Supplementary/EdanzEditingCertificate.pdf]

理文编辑

# Certificate of Editing

Edited provisional title
The MIR-Domain of PbbHLH2 is Involved in Regulating the Anthocyanin Biosynthetic Pathway of ‘Red Zaosu’ (Pyrus bretschneideri Rehd.) Pear Fruit

Client name and institution
Xieyu Li, College of Horticulture, Northwest A&F University, Taicheng Road NO.3, Yangling, Shanxi Province, China

Date Completed
2021-02-24

Identification code
94243

Certificate issued by
Koji Yamashita
Managing Director and CEO

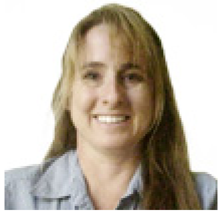

Expert Editor: Lesley Benyon
1999 PhD Genetics and Molecular Biology
University of North Carolina
Biochemistry and Cell Biology, Genetics, Plant Biology

www.liwenbianji.cn
While this certificate confirms the authors have used Edanz's editing services, we cannot guarantee that additional changes have not been made after our edits.
